# 5-Amino-2-methyl­benzene­sulfonamide

**DOI:** 10.1107/S1600536809026142

**Published:** 2009-07-11

**Authors:** Xiang-Jun Ma, Zheng Fang, Li-Li Ren, Ping Wei

**Affiliations:** a School of Biotechnology and Pharmaceutical Engineering, Nanjing University of Technology, Nanjing 210009, People’s Republic of China; b School of Pharmaceutical Sciences, Nanjing University of Technology, Nanjing 210009, People’s Republic of China

## Abstract

In the crystal structure of the title compound, C_7_H_10_N_2_O_2_S, a benzoic acid derivative, inter­molecular N—H⋯O inter­actions link the mol­ecules into a three-dimensional network.

## Related literature

For bond-length data, see: Allen *et al.* (1987 ▶).
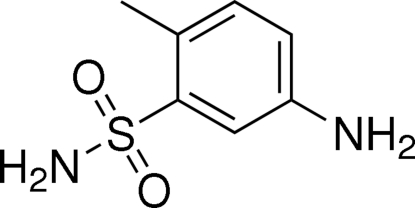


## Experimental

### 

#### Crystal data

C_7_H_10_N_2_O_2_S
*M* *_r_* = 186.23
Orthorhombic, 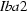

*a* = 10.679 (2) Å
*b* = 22.431 (5) Å
*c* = 7.1980 (14) Å
*V* = 1724.2 (6) Å^3^
*Z* = 8
Mo *K*α radiation
μ = 0.34 mm^−1^
*T* = 294 K
0.30 × 0.20 × 0.10 mm

#### Data collection

Enraf–Nonius CAD-4 diffractometer
Absorption correction: ψ scan (North *et al.*, 1968 ▶) *T*_min_ = 0.906, *T*_max_ = 0.967
1587 measured reflections
1432 independent reflections
1369 reflections with *I* > 2σ(*I*)
*R*_int_ = 0.044
3 standard reflections frequency: 120 min intensity decay: 1%

#### Refinement

*R*[*F* ^2^ > 2σ(*F* ^2^)] = 0.040
*wR*(*F* ^2^) = 0.120
*S* = 1.00
1432 reflections
111 parameters
1 restraint
H-atom parameters constrained
Δρ_max_ = 0.29 e Å^−3^
Δρ_min_ = −0.35 e Å^−3^
Absolute structure: Flack (1983 ▶), 576 Friedel pairs
Flack parameter: 0.04 (14)

### 

Data collection: *CAD-4 Software* (Enraf–Nonius, 1989 ▶); cell refinement: *CAD-4 Software*; data reduction: *XCAD4* (Harms & Wocadlo, 1995 ▶); program(s) used to solve structure: *SHELXS97* (Sheldrick, 2008 ▶); program(s) used to refine structure: *SHELXL97* (Sheldrick, 2008 ▶); molecular graphics: *ORTEP-3 for Windows* (Farrugia, 1997 ▶) and *PLATON* (Spek, 2009 ▶); software used to prepare material for publication: *SHELXL97*.

## Supplementary Material

Crystal structure: contains datablocks global, I. DOI: 10.1107/S1600536809026142/hk2722sup1.cif

Structure factors: contains datablocks I. DOI: 10.1107/S1600536809026142/hk2722Isup2.hkl

Additional supplementary materials:  crystallographic information; 3D view; checkCIF report

## Figures and Tables

**Table 1:** Hydrogen-bond geometry (Å, °)

*D*—H⋯*A*	*D*—H	H⋯*A*	*D*⋯*A*	*D*—H⋯*A*
N1—H1*B*⋯O2^i^	0.86	2.27	2.996 (4)	142
N1—H1*C*⋯O1^ii^	0.86	2.16	3.001 (4)	164
N2—H2*C*⋯O1^iii^	0.86	2.60	3.278 (4)	137

Symmetry codes: (i) 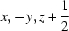; (ii) 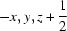; (iii) 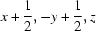.

**Fig. 1.**
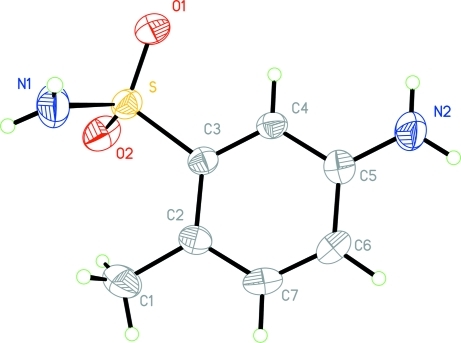
The molecular structure of the title molecule, with the atom-numbering scheme. Displacement ellipsoids are drawn at the 30% probability level.

**Fig. 2.**
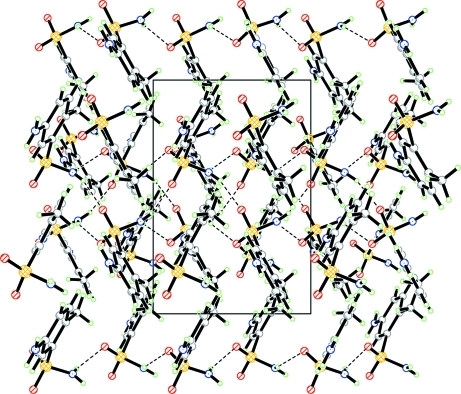
A partial packing diagram of the title compound. Hydrogen bonds are shown as dashed lines.

## supplementary crystallographic information

### Comment

Some derivatives of benzoic acid are important chemical materials. We report herein the crystal structure of the title compound.

In the molecule of the title compound, (Fig. 1), the bond lengths (Allen *et al.*,  1987) and angles are within normal ranges. Ring A (C2-C7) is, of course, planar. Atoms S, O1, N2 and C1 are 0.013 (3), -0.102 (3), -0.027 (3) and -0.032 (3) Å away from the plane of ring A, respectively.

In the crystal structure, intermolecular N-H···O interactions (Table 1) link the molecules into a three-dimensional network (Fig. 2), in which they may be effective in the stabilization of the structure.

### Experimental

For the preparation of the title compound, ammonium hydroxide (25 ml) was added to *N*-acetylamino-2-toluenesulfonyl chloride (10.7 g). The mixture was cooled down, and sulfuric acid solution (10 ml, 20%) was slowly added. The mixture was kept at 273–278 K for 5 min. The corresponding sulfonamide was collected, washed with ice water and dried to give a crystalline crude colorless solid  (yield; 67%). Then, hydrochloric acid (15 ml, 18%) was added to *N*-acetyltoluenesulfonamide (5.6 g) and the mixture was refluxed for 20 min. The resulting solution was diluted with an equal volume of water and sodium carbonate until pH = 8. After cooling, the precipitate was collected and washed with ice water (yield; 3.9 g). Crystals suitable for X-ray analysis were obtained by slow evaporation of an ethanol solution.

### Refinement

H atoms were positioned geometrically, with N-H = 0.86 Å (for NH_2_) and C-H = 0.93 and 0.96 Å for aromatic and methyl H, respectively, and constrained to ride on their parent atoms, with U_iso_(H) = xU_eq_(C,N), where x = 1.5 for methyl H and x = 1.2 for all other H atoms.

### Crystal data

**Table:**

C_7_H_10_N_2_O_2_S	*F*(000) = 784
*M**_r_* = 186.23	*D*_x_ = 1.435 Mg m^−^^3^
Orthorhombic, *I**b**a*2	Mo *K*α radiation, λ = 0.71073 Å
Hall symbol: I 2 -2c	Cell parameters from 25 reflections
*a* = 10.679 (2) Å	θ = 10–14°
*b* = 22.431 (5) Å	µ = 0.34 mm^−^^1^
*c* = 7.1980 (14) Å	*T* = 294 K
*V* = 1724.2 (6) Å^3^	Block, colorless
*Z* = 8	0.30 × 0.20 × 0.10 mm

### Data collection

**Table:**

Enraf–Nonius CAD-4 diffractometer	1369 reflections with *I* > 2σ(*I*)
Radiation source: fine-focus sealed tube	*R*_int_ = 0.044
graphite	θ_max_ = 25.3°, θ_min_ = 1.8°
ω/2θ scans	*h* = −12→0
Absorption correction: ψ scan (North *et al.*, 1968)	*k* = −26→8
*T*_min_ = 0.906, *T*_max_ = 0.967	*l* = −8→8
1587 measured reflections	3 standard reflections every 120 min
1432 independent reflections	intensity decay: 1%

### Refinement

**Table:**

Refinement on *F*^2^	Hydrogen site location: inferred from neighbouring sites
Least-squares matrix: full	H-atom parameters constrained
*R*[*F*^2^ > 2σ(*F*^2^)] = 0.040	*w* = 1/[σ^2^(*F*_o_^2^) + (0.1*P*)^2^ + 0.35*P*] where *P* = (*F*_o_^2^ + 2*F*_c_^2^)/3
*wR*(*F*^2^) = 0.120	(Δ/σ)_max_ < 0.001
*S* = 1.00	Δρ_max_ = 0.28 e Å^−^^3^
1432 reflections	Δρ_min_ = −0.35 e Å^−^^3^
111 parameters	Extinction correction: *SHELXL97* (Sheldrick, 2008), Fc^*^=kFc[1+0.001xFc^2^λ^3^/sin(2θ)]^-1/4^
1 restraint	Extinction coefficient: 0.019 (2)
Primary atom site location: structure-invariant direct methods	Absolute structure: Flack (1983), 576 Friedel pairs
Secondary atom site location: difference Fourier map	Flack parameter: 0.04 (14)

### Special details

**Table:**

Geometry. All e.s.d.'s (except the e.s.d. in the dihedral angle between two l.s. planes) are estimated using the full covariance matrix. The cell e.s.d.'s are taken into account individually in the estimation of e.s.d.'s in distances, angles and torsion angles; correlations between e.s.d.'s in cell parameters are only used when they are defined by crystal symmetry. An approximate (isotropic) treatment of cell e.s.d.'s is used for estimating e.s.d.'s involving l.s. planes.
Refinement. Refinement of *F*^2^ against ALL reflections. The weighted *R*-factor *wR* and goodness of fit *S* are based on *F*^2^, conventional *R*-factors *R* are based on *F*, with *F* set to zero for negative *F*^2^. The threshold expression of *F*^2^ > σ(*F*^2^) is used only for calculating *R*-factors(gt) *etc*. and is not relevant to the choice of reflections for refinement. *R*-factors based on *F*^2^ are statistically about twice as large as those based on *F*, and *R*- factors based on ALL data will be even larger.

### Fractional atomic coordinates and isotropic or equivalent isotropic displacement parameters (Å^2^)

**Table:**

	*x*	*y*	*z*	*U*_iso_*/*U*_eq_	
S	0.16988 (6)	0.09021 (3)	0.64671 (14)	0.0332 (3)	
O1	0.06327 (19)	0.12096 (9)	0.5690 (4)	0.0469 (6)	
N1	0.1216 (3)	0.05631 (12)	0.8282 (5)	0.0495 (8)	
H1B	0.1362	0.0189	0.8419	0.059*	
H1C	0.0810	0.0755	0.9123	0.059*	
C1	0.4280 (3)	0.06855 (16)	0.8644 (6)	0.0527 (9)	
H1D	0.5127	0.0686	0.9089	0.079*	
H1E	0.4210	0.0420	0.7604	0.079*	
H1F	0.3730	0.0555	0.9618	0.079*	
O2	0.2333 (2)	0.04672 (10)	0.5348 (4)	0.0511 (7)	
N2	0.3024 (3)	0.30886 (10)	0.6676 (5)	0.0489 (8)	
H2B	0.2329	0.3169	0.6125	0.059*	
H2C	0.3532	0.3372	0.6962	0.059*	
C2	0.3922 (2)	0.13040 (13)	0.8053 (4)	0.0316 (6)	
C3	0.2794 (2)	0.14549 (11)	0.7132 (4)	0.0272 (6)	
C4	0.2507 (2)	0.20373 (11)	0.6649 (5)	0.0287 (6)	
H4A	0.1767	0.2118	0.6017	0.034*	
C5	0.3323 (3)	0.25064 (13)	0.7103 (5)	0.0318 (7)	
C6	0.4439 (3)	0.23609 (14)	0.8042 (5)	0.0357 (7)	
H6A	0.4997	0.2662	0.8367	0.043*	
C7	0.4711 (3)	0.17810 (14)	0.8483 (4)	0.0363 (7)	
H7A	0.5458	0.1701	0.9098	0.044*	

### Atomic displacement parameters (Å^2^)

**Table:**

	*U*^11^	*U*^22^	*U*^33^	*U*^12^	*U*^13^	*U*^23^
S	0.0294 (4)	0.0314 (4)	0.0390 (5)	−0.0021 (2)	−0.0014 (3)	−0.0050 (3)
O1	0.0350 (11)	0.0441 (12)	0.0615 (15)	−0.0049 (10)	−0.0151 (11)	0.0014 (11)
N1	0.0459 (16)	0.0367 (14)	0.066 (2)	0.0010 (12)	0.0155 (16)	0.0131 (13)
C1	0.0487 (19)	0.0423 (17)	0.067 (2)	0.0114 (15)	−0.016 (2)	0.0051 (18)
O2	0.0447 (14)	0.0482 (13)	0.0603 (17)	−0.0016 (10)	0.0036 (12)	−0.0242 (12)
N2	0.0561 (15)	0.0312 (12)	0.059 (2)	−0.0070 (11)	−0.0146 (18)	0.0069 (14)
C2	0.0283 (14)	0.0348 (15)	0.0317 (15)	0.0046 (11)	0.0002 (12)	−0.0003 (11)
C3	0.0236 (12)	0.0307 (13)	0.0273 (13)	0.0010 (10)	0.0024 (11)	−0.0021 (11)
C4	0.0249 (12)	0.0330 (13)	0.0281 (15)	−0.0009 (10)	−0.0035 (12)	0.0019 (11)
C5	0.0374 (14)	0.0322 (14)	0.0258 (14)	0.0000 (10)	0.0029 (12)	0.0010 (11)
C6	0.0309 (13)	0.0422 (16)	0.0339 (15)	−0.0101 (12)	−0.0014 (13)	−0.0054 (13)
C7	0.0248 (14)	0.0503 (17)	0.0338 (16)	0.0022 (11)	−0.0052 (12)	−0.0034 (11)

### Geometric parameters (Å, °)

**Table:**

S—O2	1.435 (2)	N2—H2B	0.8600
S—O1	1.444 (2)	N2—H2C	0.8600
S—N1	1.597 (3)	C2—C7	1.397 (4)
S—C3	1.770 (3)	C2—C3	1.416 (4)
N1—H1B	0.8600	C3—C4	1.386 (4)
N1—H1C	0.8600	C4—C5	1.405 (4)
C1—C2	1.501 (4)	C4—H4A	0.9300
C1—H1D	0.9600	C5—C6	1.408 (4)
C1—H1E	0.9600	C6—C7	1.370 (5)
C1—H1F	0.9600	C6—H6A	0.9300
N2—C5	1.379 (4)	C7—H7A	0.9300
			
O2—S—O1	118.64 (18)	C7—C2—C3	115.7 (3)
O2—S—N1	106.75 (16)	C7—C2—C1	119.5 (3)
O1—S—N1	106.85 (15)	C3—C2—C1	124.8 (3)
O2—S—C3	108.43 (14)	C4—C3—C2	122.1 (2)
O1—S—C3	106.92 (12)	C4—C3—S	116.5 (2)
N1—S—C3	108.98 (17)	C2—C3—S	121.4 (2)
S—N1—H1B	120.0	C3—C4—C5	120.7 (3)
S—N1—H1C	120.0	C3—C4—H4A	119.7
H1B—N1—H1C	120.0	C5—C4—H4A	119.7
C2—C1—H1D	109.5	N2—C5—C4	120.9 (3)
C2—C1—H1E	109.5	N2—C5—C6	121.5 (3)
H1D—C1—H1E	109.5	C4—C5—C6	117.6 (3)
C2—C1—H1F	109.5	C7—C6—C5	120.7 (3)
H1D—C1—H1F	109.5	C7—C6—H6A	119.7
H1E—C1—H1F	109.5	C5—C6—H6A	119.7
C5—N2—H2B	120.0	C6—C7—C2	123.3 (3)
C5—N2—H2C	120.0	C6—C7—H7A	118.4
H2B—N2—H2C	120.0	C2—C7—H7A	118.4
			
C7—C2—C3—C4	1.5 (5)	C2—C3—C4—C5	−1.6 (5)
C1—C2—C3—C4	178.9 (3)	S—C3—C4—C5	−179.5 (2)
C7—C2—C3—S	179.3 (2)	C3—C4—C5—N2	−177.9 (3)
C1—C2—C3—S	−3.3 (5)	C3—C4—C5—C6	0.7 (5)
O2—S—C3—C4	123.0 (3)	N2—C5—C6—C7	178.9 (3)
O1—S—C3—C4	−6.0 (3)	C4—C5—C6—C7	0.3 (5)
N1—S—C3—C4	−121.1 (3)	C5—C6—C7—C2	−0.3 (5)
O2—S—C3—C2	−54.9 (3)	C3—C2—C7—C6	−0.6 (5)
O1—S—C3—C2	176.1 (3)	C1—C2—C7—C6	−178.1 (3)
N1—S—C3—C2	61.0 (3)		

### Hydrogen-bond geometry (Å, °)

**Table:**

*D*—H···*A*	*D*—H	H···*A*	*D*···*A*	*D*—H···*A*
N1—H1B···O2^i^	0.86	2.27	2.996 (4)	142
N1—H1C···O1^ii^	0.86	2.16	3.001 (4)	164
N2—H2C···O1^iii^	0.86	2.60	3.278 (4)	137

Symmetry codes: (i) *x*, −*y*, *z*+1/2; (ii) −*x*, *y*, *z*+1/2; (iii) *x*+1/2, −*y*+1/2, *z*.
